# Predicting acute radiation induced xerostomia in head and neck Cancer using MR and CT Radiomics of parotid and submandibular glands

**DOI:** 10.1186/s13014-019-1339-4

**Published:** 2019-07-29

**Authors:** Khadija Sheikh, Sang Ho Lee, Zhi Cheng, Pranav Lakshminarayanan, Luke Peng, Peijin Han, Todd R. McNutt, Harry Quon, Junghoon Lee

**Affiliations:** Department of Radiation Oncology and Molecular Radiation Sciences, Johns Hopkins University School of Medicine, 401 North Broadway, Suite 1440, Baltimore, MD 21287-5678 USA

**Keywords:** Head and neck cancer, Radiation therapy, Radiomics, Machine learning, Xerostomia

## Abstract

**Purpose:**

To analyze baseline CT/MR-based image features of salivary glands to predict radiation-induced xerostomia 3-months after head-and-neck cancer (HNC) radiotherapy.

**Methods:**

A retrospective analysis was performed on 266 HNC patients who were treated using radiotherapy at our institution between 2009 and 2018. CT and T1 post-contrast MR images along with NCI-CTCAE xerostomia grade (3-month follow-up) were prospectively collected at our institution. CT and MR images were registered on which parotid/submandibular glands were contoured. Image features were extracted for ipsilateral/contralateral parotid and submandibular glands relative to the location of the primary tumor. Dose-volume-histogram (DVH) parameters were also acquired. Features were pre-selected based on Spearman correlation before modelling by examining the correlation with xerostomia (*p* < 0.05). A shrinkage regression analysis of the pre-selected features was performed using LASSO. The internal validity of the variable selection was estimated by repeating the entire variable selection procedure using a leave-one-out-cross-validation. The most frequently selected variables were considered in the final model. A generalized linear regression with repeated ten-fold cross-validation was developed to predict radiation-induced xerostomia at 3-months after radiotherapy. This model was tested in an independent dataset (*n* = 50) of patients who were treated at the same institution in 2017–2018. We compared the prediction performances under eight conditions (DVH-only, CT-only, MR-only, CT + MR, DVH + CT, DVH + CT + MR, Clinical+CT + MR, and Clinical+DVH + CT + MR) using the area under the receiver operating characteristic curve (ROC-AUC).

**Results:**

Among extracted features, 7 CT, 5 MR, and 2 DVH features were selected. The internal cohort (*n* = 216) ROC-AUC values for DVH, CT, MR, and Clinical+DVH + CT + MR features were 0.73 ± 0.01, 0.69 ± 0.01, 0.70 ± 0.01, and 0.79 ± 0.01, respectively. The validation cohort (*n* = 50) ROC-AUC values for DVH, CT, MR, and Clinical+DVH + CT + MR features were 0.63, 0.57, 0.66, and 0.68, respectively. The DVH-ROC was not significantly different than the CT-ROC (*p* = 0.8) or MR-ROC (*p* = 0.4). However, the CT + MR-ROC was significantly different than the CT-ROC (*p* = 0.03), but not the Clinical+DVH + CT + MR model (*p* = 0.5).

**Conclusion:**

Our results suggest that baseline CT and MR image features may reflect baseline salivary gland function and potential risk for radiation injury. The integration of baseline image features into prediction models has the potential to improve xerostomia risk stratification with the ultimate goal of truly personalized HNC radiotherapy.

**Electronic supplementary material:**

The online version of this article (10.1186/s13014-019-1339-4) contains supplementary material, which is available to authorized users.

## Background

Radiation therapy (RT), often with concurrent chemotherapy, is frequently used in the management of head and neck cancer (HNC) as definitive or adjuvant treatment. RT for HNC improves local control but is associated with significant treatment-related toxicities such as xerostomia [1, 2]. Approximately 50–80% of patients with HNC will experience xerostomia to some degree after RT [3, 4]. While these swallow-related toxicities significantly influence long-term patient outcomes and quality of life, our ability to robustly characterize these complications as they relate to individual patients and the radiotherapy dosimetry delivered to salivary glands is limited.

In radiation oncology, there is increasing popularity for rapid-learning health systems which use routine clinical data to develop models that can be used to predict patient specific treatment outcomes [5–7]. In addition to predicting outcomes, the goal of decision support systems is to improve overall patient care and determine when and how to personalize patients’ treatments. Machine learning algorithms have emerged as popular tools for decision support. These algorithms are already being applied to many aspects of radiation therapy including: target delineation [8, 9], treatment planning [10, 11], radiation physics quality assurance [12], and outcome [13] and tumor response modelling [14]. With recent advancements in image processing, informatics, and machine learning, medical imaging is increasingly being used for improved clinical decision making. Studies have demonstrated that the variability in clinical image intensity, shape, and texture can be quantified generating a radiomic signature for individual tumors and normal anatomic structures [15–20]. For radiation therapy, radiomics offers the potential to significantly influence clinical decision-making, therapy planning, and follow-up workflow. In HNC, a radiomic signature has been shown to be prognostic and has been validated across several institutions [19, 20]. Radiomics derived from computed tomography (CT) have also been used to predict xerostomia and survival in HNC patients [18, 21, 22].

To our knowledge, the incorporation of MR-based biomarkers with CT and dosimetry features in acute RT-induced xerostomia prediction models has not been investigated in HNC. Thus, the objective of this study was to analyze baseline CT/MR image features of salivary glands to better understand their role in the prediction of radiation-induced xerostomia 3 months after HNC radiotherapy. We hypothesized that baseline CT/MR image features are related to xerostomia and incorporating these into a prediction model improves the accuracy of predicting radiation-induced xerostomia compared to dosimetric information alone.

## Materials and methods

### Patients

HNC patients treated at Johns Hopkins Hospital who underwent intensity modulated radiotherapy (IMRT) from 2009 through 2018 (on a protocol for retrospective data analysis approved by the institutional review board) were included. Patients who did not have MR images were excluded. NCI-CTCAE v4.0 xerostomia grade was assessed by physicians at the point of care at 3 months post-RT. Moderate to severe xerostomia incidence was defined as grade ≥ 2, compared to the reference group which was defined as xerostomia grade 0 or 1. All patients were treated with either IMRT, VMAT, or TomoTherapy. All treatments included a simultaneous integrated boost and attempted to spare dose to the parotid glands and swallowing structures without compromising the dose to the target volumes.

### Image data

All images were acquired at the time of simulation, prior to the start of treatment. For both training and validation sets, T1-weighted MRI was acquired using Siemens Magnetom Espree 1.5 T scanner (Siemens Medical Systems, Erlangen, Germany) with a turbo spin echo sequence post-Gd administration (TE = 8.9 ms, TR = 577 ms, flip angle = 150°, matrix size = 256 × 256, pixel size ranged from 0.8 × 0.8 mm^2^ to 1.1 × 1.1 mm^2^ depending on the field of view defined at simulation, and slice thickness = 3 mm). To reduce bias and improve interpretation of the image features, MR images were resampled such that the in-plane pixel size was consistently 0.89 × 0.89 mm^2^, which was the size for majority of the patients. CT images were acquired using a 16-slice Philips Brilliance Big Bore scanner (Philips, Andover, MA) with tube voltage 120 kVp and exposure of 200 mAs. Images had 512 × 512 pixels with a pixel size of 1.2 × 1.2 mm^2^, and a slice thickness of 3 mm. CT images with metal artifacts (most commonly caused by dental fillings and implants) were corrected using Metal Artifact Reduction for Orthopedic Implants reconstruction. However, patients with severe artifacts were excluded in the image analysis to avoid undesirable strong influence to the image features and analysis.

### Feature extraction

For each patient, ipsilateral/contralateral parotid and submandibular glands (iPG, cPG, iSG, cSG) were contoured by the attending radiation oncologist. The salivary gland volumes (including combined salivary gland volumes) were determined in centimeters cubed. The tumor volume was also determined for each patient in centimeters cubed. Missing contours were automatically segmented using in-house implemented multi-atlas-based auto-segmentation software based on a GPU-accelerated demons deformable image registration [23] and a statistical label fusion [24]. Each auto-segmented contour was visually checked and manually corrected for any erroneously segmented regions. CT images were registered to the MR images using Velocity (V3.2.1, Varian Medical Systems Inc., Palo Alto, CA). Contours were propagated from CT images to the MR images. Each co-registration was visually verified by overlapping the registered CT and the target MR images focusing on the target glands as well as by overlapping the propagated contours on the target by a single observer (KS). For each derived region of interest (ROI), dose-volume histograms (DVHs) features were calculated in 5% increments from D10 to D95.

**Fig. 1:**
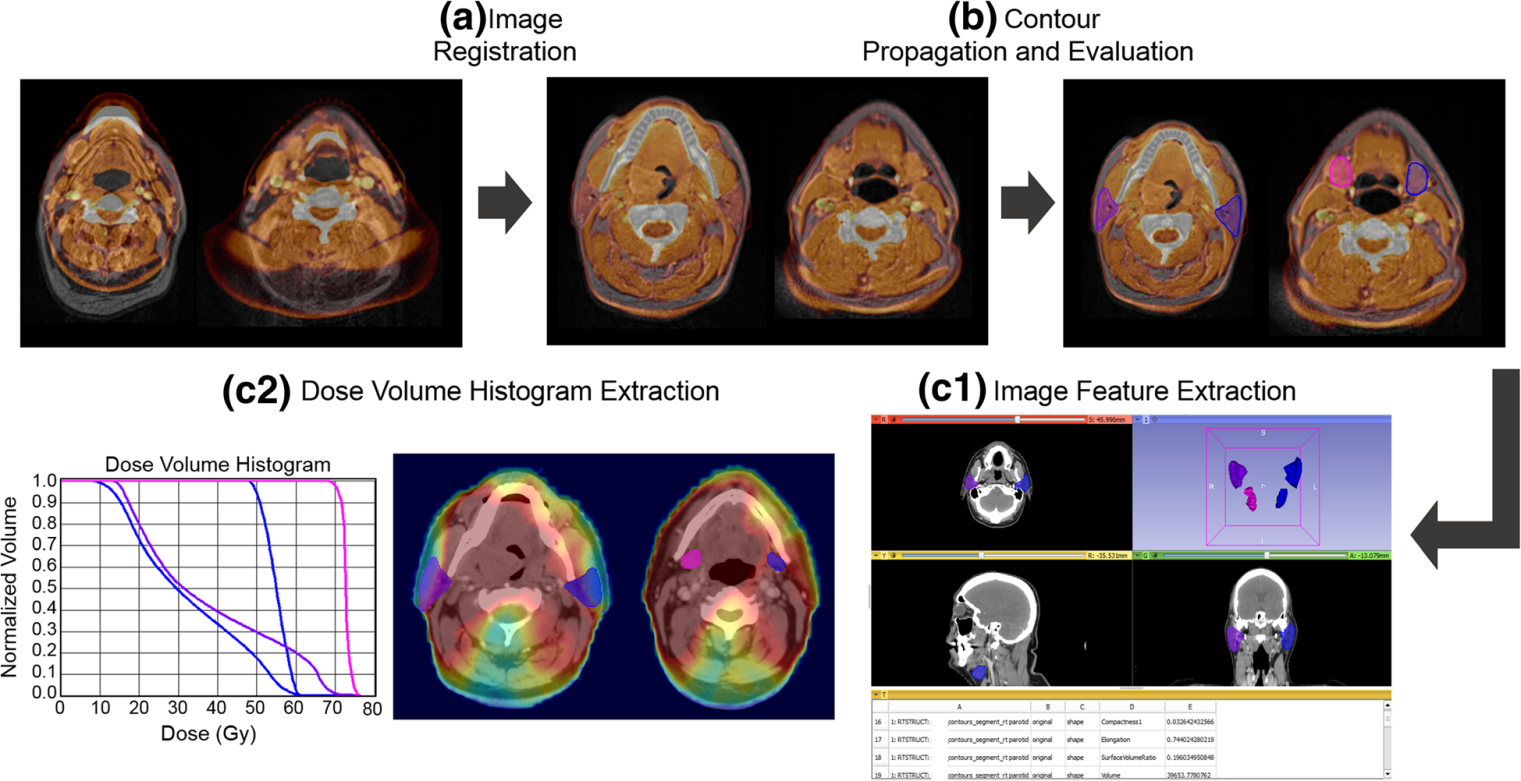
Radiomics feature extraction pipeline. **a** CT images (shown in hot color map) were registered to the MR images (shown in grayscale). Salivary gland contours were propagated from CT images to the MR images (**b**). For each segmented salivary gland ROI, high-dimensional image features were extracted from both CT and MR images (**c1**), and DVH features (**c2**)

For each segmented salivary gland ROI, high-dimensional image features were extracted from both CT and MR images using the *PyRadiomics* software package accessed via the Radiomics module of 3D Slicer [25]. The ROI was analyzed as a 3D volume. A schematic showing the feature extraction process is shown in Fig. 1. CT gray level intensities were discretized at a bin width of 25. This fixed bin width resulted in 20–25 bins for CT images based on the salivary gland ROI specified. It should be noted that texture features have been shown to be affected by the bin width used to discretize image intensities [26]. Although the optimal bin width for image feature analysis has not been established, previous HNC work has used a 25 unit bin width for the evaluation of image features [27, 28]. A fixed bin count of 25 was used for the MR images as per the Image Biomarker Standardization Initiative guidelines [29]. Briefly, a fixed bin count introduces a normalizing effect for MR which may be beneficial when intensity units are arbitrary and allows for a direct comparison of feature values across multiple analyzed ROIs (e.g. across different samples). All textural features were normalized by subtracting the values from their mean and dividing by the standard deviation.

Major categories of extracted features included shape, first order statistics, gray level co-occurrence matrix (GLCM), gray level run-length matrix (GLRLM), and gray level size-zone matrix (GLSZM) features derived from original images as well as after wavelet filtering. The angles required for the computation of the GLCM and GLRLM were automatically generated and averaged to achieve rotational invariance. The distance for the associated angle was set to 1 voxel in all directions for the GLCM. We used 25 equally sized bins for CT and 25 fixed bin count for MR for first-order statistics, and gray levels were quantized into 20–25 levels for CT and 25 levels for MR for GLCM and GLRLM calculations. For the detailed list of features calculated, we refer readers to [25]. Wavelet filtering resulted in either a high-pass or low-pass filter in each of the three dimensions (e.g. wavelet LLL corresponded to low-pass filter applied in the x-, y-, and z-axis directions). In broad terms, first order features describe the statistics of voxel intensity distributions within the ROI while higher order features such as GLCM, GLRLM, and GLSZM seek to quantify textural characteristics. Taken together, 2877 image features and 48 DVH features were extracted for each patient. The datasets used and analyzed during the current study are available from the corresponding author on reasonable request.

### Feature selection and xerostomia prediction

As per Transparent Reporting of a multivariable prediction model for Individual Prognosis Or Diagnosis (TRIPOD) guidelines [30], a Spearman correlation was used prior to modelling to pre-select features that were correlated with the outcome (*p* < 0.05). There was no correction performed for multiple testing. This resulted in 102 image features and 44 DVH features. A shrinkage regression analysis of the pre-selected features (including the image features, DVH information, age, gender, and tumor volume) was performed using the least absolute shrinkage and selection operator (LASSO). The internal validity of the variable selection was estimated by repeating the entire variable selection procedure using a leave-one-out-cross-validation. The most frequently selected variables (> 50%) were considered in the final model. Finally, to further address collinearity, if the correlation coefficient between two features was larger than 0.80, only the variable with the highest correlation with xerostomia was selected, as previously described [31, 32]. This resulted in one image feature to be removed (specifically, the CT contralateral submandibular wavelet LLH GLSZM Gray Level Non Uniformity Normalized). Feature selection was performed on the training set only (specifically, patients treated between the years 2009 and 2016).

Trained on the institution cohort of patients treated between the years 2009 and 2016, prediction modelling was performed using generalized linear model (GLM) with a repeated ten-fold cross validation [33] to predict radiation-induced xerostomia at 3 months after RT. The ten-fold cross validation was performed 100 times with random initialization of 10 disjoint groups. As per TRIPOD guidelines [30], in an independent set of patients who were treated in 2017 and 2018 (specifically after December 31, 2016), we compared the prediction performance under eight different scenarios: 1) only DVH features, 2) only CT image features, 3) only MR image features, 4) both CT and MR image features, 5) DVH and CT image features, 6) DVH and CT/MR image features, 7) clinical and CT/MR image features, and 8) clinical, DVH, CT/MR image features. Clinical data included age, sex, and tumor volume. The model performance measures were the area under the receiver operating characteristic curve (ROC-AUC). DeLong’s test was used to analyze the areas under correlated ROC curves [34]. The 95% confidence interval (CI) was computed for the AUC.

All statistical analysis and predictive modeling was performed in R (version 3.4.1). Results were considered significant when the probability of making a Type I error was less than 5% (*p* < 0.05).

## Results

### Study subjects

Two hundred and sixty-six HNC patients were evaluated including those with and without xerostomia. Table 1 shows demographics, tumor and DVH information for all patients. Most patients had tumors of the oropharynx (*n* = 119/266, 45%) or the oral cavity (*n* = 23/266, 23%). All patients were treated with IMRT, specifically, 7% of patients in the training cohort and 44% of patients in the validation cohort, received TomoTherapy.

**Table 1:** Characteristics for head and neck cancer patients (*n* = 266). Continuous variables are displayed as mean (SD), while categorical variables are displayed as count (%)

	Internal Validation Cohort (n = 216)	External Validation Cohort (n = 50)
Patient Demographics		
Age (yrs)	58 (10)	60 (13)
Male n	176 (81)	46 (92)
Xerostomia > 2 n	87 (40)	14 (28)
TomoTherapy n	16 (7)	22 (44)
Tumor Site		
Oropharynx	92 (43)	27 (54)
Oral Cavity	49 (23)	13 (26)
Nasopharynx	13 (6)	6 (12)
Hypopharynx	4 (2)	0 (0)
Larynx	19 (9)	3 (6)
Thyroid	5 (2)	0 (0)
Accessory Sinuses	7 (3)	1 (2)
Other	27 (12)	0 (0)
DVH Parameters		
cPG D40 (Gy)	27.8 (14.7)	22.8 (9.9)
cSG D60 (Gy)	56.8 (23.5)	49.5 (17.8)

**Fig. 2:**
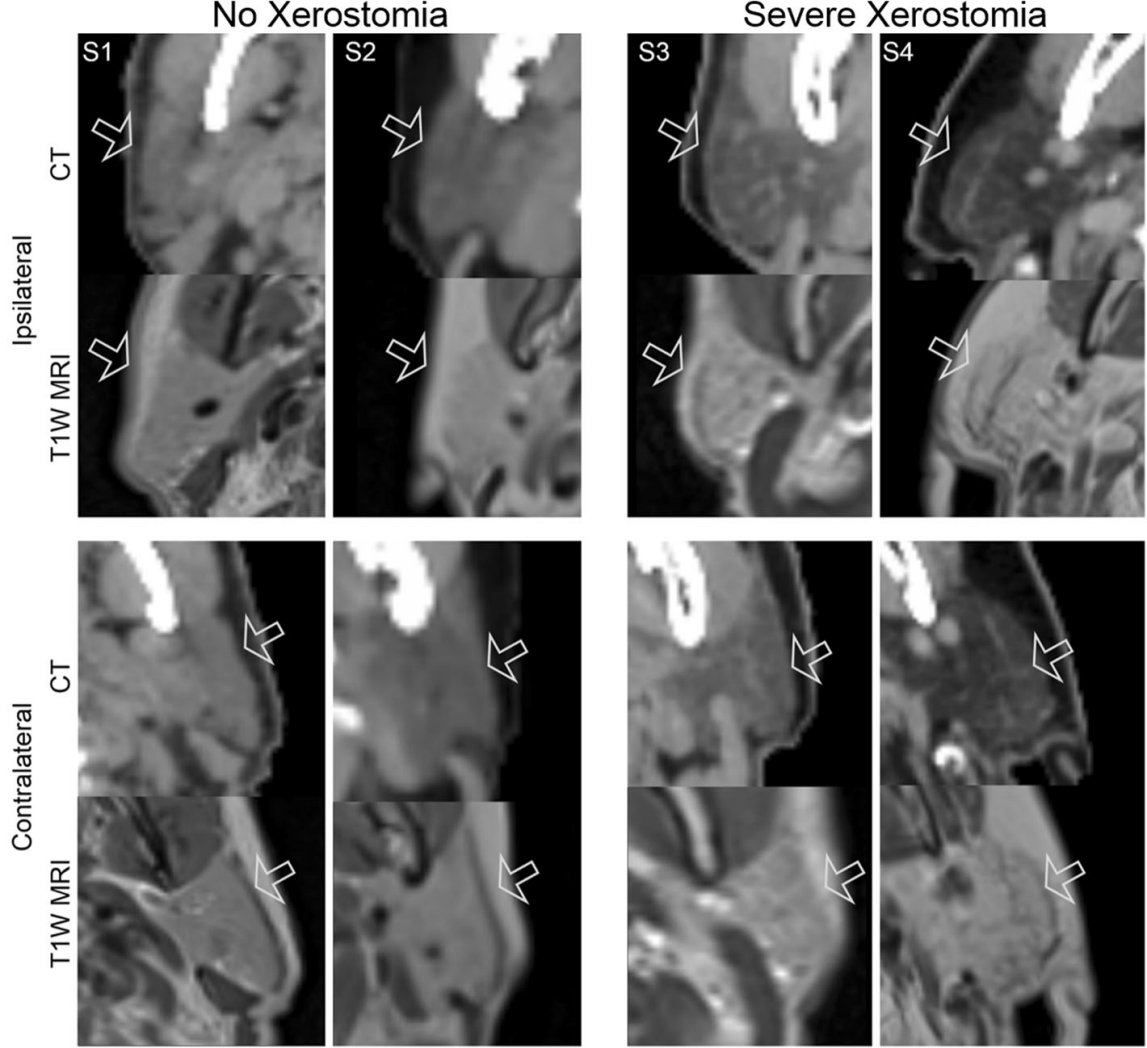
CT and MR images of representative patients’ parotid glands. From left to right: S1: 57-yr old male with squamous cell carcinoma of the nasopharynx, S2: 82-yr old female with melanoma of nasal cavity, S3: 61-yr old male with squamous cell carcinoma of the nasopharynx, and S4: 69-yr old male with carcinoma ex pleomorphic adenoma of eye. Images are displayed using the same window and level

CT and MR images of parotid glands from four representative patients are shown in Fig. 2. Briefly, patients with post-treatment xerostomia appeared to have more hypodense and heterogeneous parotid glands at baseline, compared to those without xerostomia in the CT images. Both CT and MR images of patients with xerostomia appeared to have more regions of lower intensity.

**Fig. 3:**
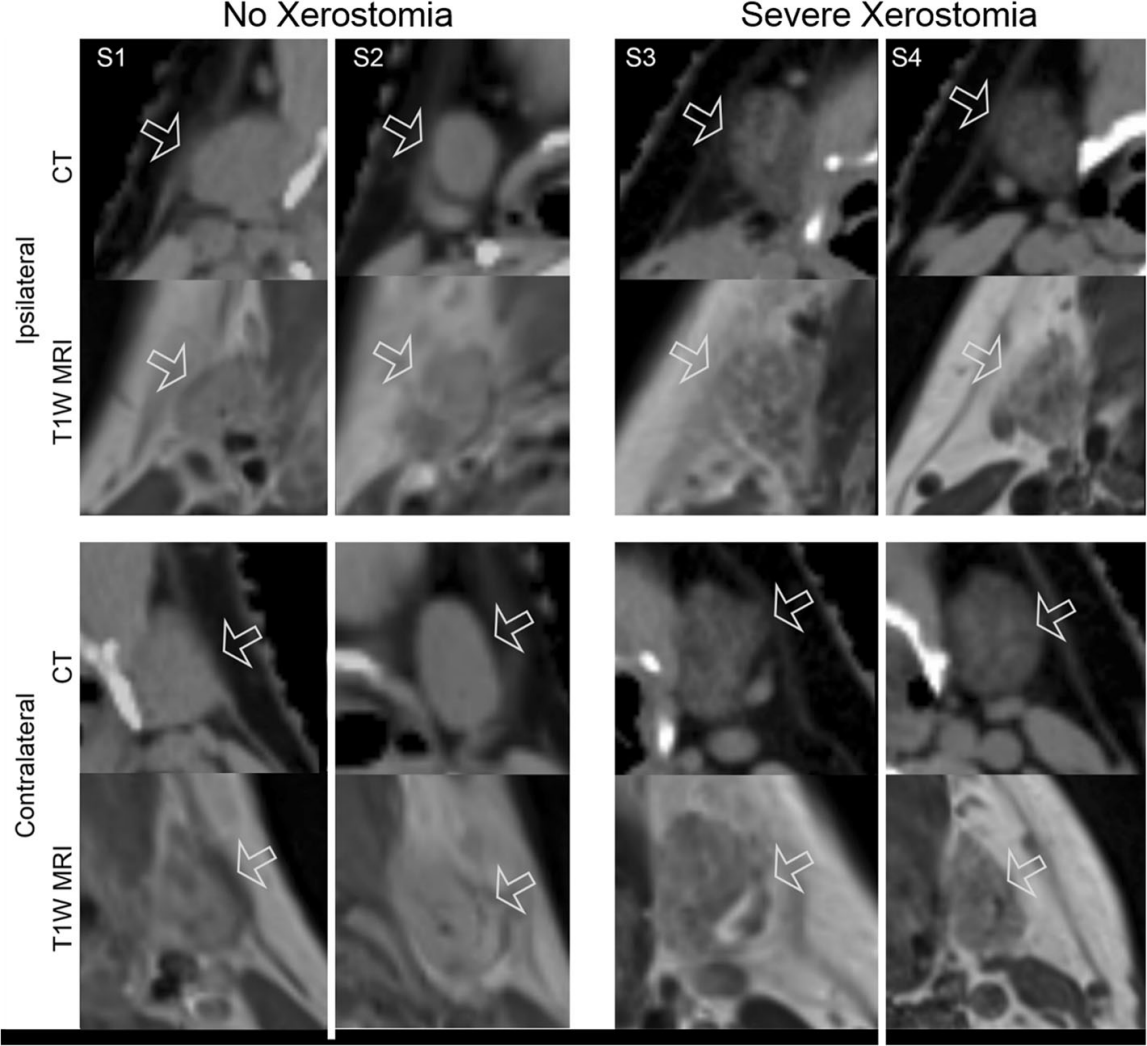
CT and MR images of representative patients’ submandibular glands. From left to right: S1: 71-yr old male with squamous cell carcinoma of the base of tongue, S2: 82-yr old female with melanoma of nasal cavity, S3: 54-yr old female with squamous cell carcinoma of the tonsil, and S4: 61-yr old male with squamous cell carcinoma of the nasopharynx. Images are displayed using the same window and level

Figure 3 shows CT and MR images of submandibular glands for four representative patients. On CT, the submandibular glands of patients with xerostomia again appeared more hypodense and heterogeneous compared to those patients without xerostomia. The MR images of patients with xerostomia appear to be more heterogeneous and hypointense than the patients without xerostomia similarly as in CT.

### Xerostomia prediction models

Table 2 shows the performance of prediction models for development of xerostomia at 3 months post-RT for the internal validation cohort. Using DeLong’s test, the DVH-ROC was not significantly different from MR-ROC (*p* = 0.6) and CT-ROC (*p* = 0.6). The CT-ROC was not significantly different from the MR-ROC (*p* = 0.7). Combining CT with MR features suggested an improvement in xerostomia prediction performance compared to CT features alone (*p* = 0.01). Finally, the model performance improved with the combination of DVH and CT + MR features compared to DVH (*p* = 0.003) or CT + MR (*p* = 0.03). There was a trend towards significance when comparing the DVH + CT-ROC to the DVH + CT + MR-ROC (*p* = 0.06). The model performance improved with the combination of DVH and CT + MR features compared to DVH, CT, or MR alone (*p* < 0.005). Finally, adding the clinical data did not significantly change the DVH + CT + MR-ROC (*p* = 0.2). Training set ROC curves are shown in the supplement as Additional file 1: Figure S1.

**Table 2:** Multiple Logistic Regression performances using a 10-fold cross validation at predicting xerostomia at 3 months after radiotherapy for internal validation cohort (n = 216). Mean and standard deviation of area-under-the curve is reported for the repeated 10 fold cross-validation

Mean ± SD	AUC	Sensitivity	Specificity
*Generalized Linear Model*			
DVH	0.73 ± 0.01	0.76 ± 0.01	0.56 ± 0.02
CT	0.69 ± 0.01	0.76 ± 0.01	0.50 ± 0.02
MR	0.70 ± 0.01	0.80 ± 0.01	0.50 ± 0.02
CT + MR	0.75 ± 0.01	0.76 ± 0.01	0.58 ± 0.02
DVH + CT	0.77 ± 0.01	0.79 ± 0.01	0.60 ± 0.02
DVH + CT + MR	0.79 ± 0.01	0.78 ± 0.01	0.65 ± 0.02
Clinical+CT + MR	0.77 ± 0.01	0.79 ± 0.02	0.61 ± 0.02
Clinical+DVH + CT + MR	0.79 ± 0.01	0.78 ± 0.02	0.65 ± 0.02

Table 3 shows the regression coefficients (β) and odds ratios (ORs) for all the features used in the GLM prediction models. For the DVH only model, cPG D40 contributed significantly to the model (OR = 1.51, *p* < 0.001). For the CT only model, features that contributed significantly to the model included those from both salivary glands, and interestingly, most of the image features stemmed from the wavelet filtered images. For the MR only model, features from both the parotid and submandibular glands contributed significantly to the model including the cPG least axis (OR = 2.66, *p* = 0.004), iPG wavelet LHL GLSZM small area high gray level emphasis (OR = 0.50, *p* = 0.03), and iSG wavelet LHH GLSZM small area high gray level emphasis (OR = 3.22, *p* = 0.0005).

**Table 3:** GLM summary including odds ratios (OR) and 95% confidence interval (CI) for the prediction of xerostomia

	DVH Model		CT Model		MR Model		Clinical + DVH + CT + MR Model	
	β	OR (95% CI)	β	OR (95% CI)	β	OR (95% CI)	β	OR (95% CI)
Intercept	**−2.58**	**–**	**−9.21**		−0.31		**−6.54**	
Age	**–**	**–**	**–**	**–**	**–**	**–**	−0.69	0.50 (0.47–0.54)
Gender	**–**	**–**	**–**	**–**	**–**	**–**	1.09	2.97 (1.93–4.01)
Tumor Volume	**–**	**–**	**–**	**–**	**–**	**–**	0.45	1.56 (1.56–1.58)
cPG D40	**1.51**	**4.57 (4.56–4.58)**	**–**	**–**	**–**	**–**	**1.03**	**2.79 (2.78–2.79)**
cSG D60	0.60	1.81 (1.80–1.82)	**–**	**–**	**–**	**–**	0.63	1.86 (1.86–1.88)
CT cSG wavelet LLL GLSZM Gray Level Non Uniformity Normalized	**–**	**–**	**1.00**	**2.73 (−11.3–16.7)**	–	–	**0.83**	**2.29 (−14.4–19.0)**
CT iPG original GLSZM Low Gray Level Zone Emphasis	**–**	**–**	−0.64	0.53 (−9.0–10.1)	–	–	− 0.61	0.55 (−10.4–11.5)
CT iSG wavelet HLL GLCM Inverse Variance	**–**	**–**	0.57	1.77 (−17.4–20.9)	–	–	0.36	1.44 (−21.9–24.8)
CT cPG wavelet LHL Total Energy	**–**	**–**	0.42	1.53 (1.52–1.54)	–	–	0.31	1.36 (1.35–1.37)
CT iSG wavelet HLL GLRLM Long Run High Gray Level Emphasis	**–**	**–**	−0.65	0.52 (0.51–0.53)	–	–	−0.69	0.50 (0.49–0.51)
CT iPG original first order 10 Percentile	**–**	**–**	**−0.69**	**0.50 (0.49–0.51)**	–	–	−0.38	0.69 (0.68–0.70)
CT cPG wavelet LHL GLRLM Long Run High Gray Level Emphasis	**–**	**–**	0.43	1.54 (1.53–1.55)	–	–	0.49	1.63 (1.62–1.64)
MR cPG shape Least Axis Length	**–**	**–**	–	–	**0.98**	**2.66 (2.59–2.73)**	0.84	2.32 (2.23–2.42)
MR iSG wavelet LHH GLSZM Gray Level Non Uniformity Normalized	**–**	**–**	–	–	−0.65	0.52 (−26.2–27.2)	**−0.92**	**0.40 (−33.8–34.6)**
MR iSG wavelet LHH GLSZM Small Area High Gray Level Emphasis	**–**	**–**	–	–	**1.17**	**3.22 (3.20–3.23)**	**1.28**	**3.59 (3.57–3.61)**
MR iPG wavelet LHL GLSZM Small Area High Gray Level Emphasis	**–**	**–**	–	–	**−0.69**	**0.50 (0.48–0.52)**	−0.72	0.49 (0.47–0.51)
MR iSG wavelet LLH GLSZM Size Zone Non Uniformity Normalized	**–**	**–**	–	–	−0.36	0.70 (−9.75–11.2)	− 0.43	0.65 (−12.3–13.6)

*iPG* ipsilateral parotid gland, *cPG* contralateral parotid gland, *iSG* ipsilateral submandibular gland, *cSG* contralateral submandibular gland, *GLCM* gray level co-occurrence matrix, *GLSZM* gray level size zone matrix, *GLRLM* gray level run length matrix; bold indicates significant values (*p* < .05)

In the model containing Clinical, DVH, CT, and MR features, the features that significantly contributed to the model were the cPG D40 (OR = 2.79, *p* = 0.04), CT cSG wavelet LLL GLSZM gray level non uniformity normalized (OR = 2.29, *p* = 0.04), MR iSG wavelet LHH GLSZM small area high gray level emphasis (OR = 3.59, *p* = 0.002), and the MR iSG wavelet LHH GLSZM gray level non uniformity normalized (OR = 0.40, *p* = 0.04). Interestingly, all of the significant image features came from wavelet filtered images and stemmed from the GLSZM. Additional file 3: Table S1 shows the beta coefficients and OR for the CT + MR, DVH + CT, DVH + CT + MR, and Clinical+CT + MR models.

Table 4 shows the performance of the prediction models for the validation cohort and ROC curves are shown in the supplement. The CT-ROC and MR-ROC were not significantly different (*p* = 0.4). The DVH-ROC was not significantly different than the CT-ROC (*p* = 0.8) or MR-ROC (*p* = 0.4). However, the CT + MR-ROC was significantly different than the CT-ROC (*p* = 0.03), but not significantly different than the DVH-ROC (*p* = 0.4) or MR-ROC (*p* = 0.8). The Clinical+CT + MR ROC was significantly different than the CT-ROC (0.02), but not MR-ROC (0.2). The Clinical+DVH + CT + MR model was significantly different from Clinical+CT + MR (*p* = 0.03), but not from the CT + MR model (*p* = 0.5). Adding clinical data to the DVH + CT + MR model, modestly improved the model performance with a trend towards significant (*p* = 0.1). Test set ROC curves are shown in the supplement as Additional file 2: Figure S2.

**Table 4:** Multiple Logistic Regression performances at predicting xerostomia at 3 months after radiotherapy for external validation cohort (*n* = 50)

	AUC	Sensitivity	Specificity
*Generalized Linear Model*			
DVH	0.63 (0.51–0.81)	0.64	0.58
CT	0.57 (0.45–0.71)	0.50	0.68
MR	0.66 (0.54–0.82)	0.80	0.65
CT + MR	0.70 (0.57–0.82)	0.80	0.62
DVH + CT	0.56 (0.40–0.68)	0.60	0.56
DVH + CT + MR	0.60 (0.50–0.73)	0.67	0.53
Clinical+CT + MR	0.73 (0.62–0.86)	0.86	0.59
Clinical+DVH + CT + MR	0.68 (0.52–0.80)	0.67	0.68

## Discussion

In this study, to better understand the influence of image features in the prediction of RT-induced xerostomia, we investigated the relationships between CT and MR image features with xerostomia scores in HNC patients using machine learning approaches. We made the following observations: 1) image features from both the parotid and submandibular glands significantly contributed to our prediction of xerostomia, 2) higher order texture features for both ipsi- and contralateral salivary glands were important predictors of xerostomia, and 3) combining multimodal image features with dosimetry features improved xerostomia prediction. Collectively, these observations further support prior work [22, 35] demonstrating that baseline salivary gland image features with CT along with quantifying radiation injury are important in predicting for the risk of xerostomia 3 months following RT.

mage features from both salivary glands significantly contributed to the prediction of xerostomia post-RT, concordant with the readily apparent differences visualized in both salivary glands using CT and MR (Figs. 2 and 3). Patients with xerostomia after RT appeared to have more heterogeneous parotid and submandibular glands at baseline. We should note that the majority of HNC research using radiomics has focused on the parotid glands [31, 36–39] with relatively little attention paid to the submandibular glands [18, 22]. Interestingly, the features with the greatest OR corresponded to the submandibular glands. While the parotid glands produce the majority of saliva during eating and with oral stimulation, submandibular glands contribute up to more than 70% of unstimulated/resting salivary output [40] which is rich in mucin. This allows for the oral mucosa to maintain its hydration [41, 42]. These results suggest that baseline submandibular gland image features may provide insight into unstimulated salivary function, and this insight may improve prediction of susceptibility to post-RT xerostomia.

Important features in our cohort stemmed from the GLRLM and the GLSZM and both the ipsilateral and contralateral salivary glands. For the contralateral side, the CT SG wavelet LLL GLRLM gray level non-uniformity normalized significantly contributed to the GLM. The cPG CT wavelet LHL GLRLM long run high gray level emphasis, which had the second lowest standard error in the model, increases when the texture is dominated by long runs with high intensity levels. These results suggest that patients with xerostomia have cSG that have lower similarity in intensities (increased gray level non-uniformity) and more heterogeneous size zone volumes (increased size zone non-uniformity). Furthermore, patients with increased risk of xerostomia have finer structural textures of the cPG (decreased long run emphasis) [22] with longer run of high intensity voxels (increased long run high gray level emphasis). Focusing on the ipsilateral side, the feature that contributed significantly to the GLM included the MR iSG wavelet LHL GLSZM small area high gray level emphasis. This feature indicates that patients with xerostomia have ipsilateral submandibular glands with more small regions of low intensity (i.e. more locally heterogeneous as indicated by an increase of small area low gray level intensity).

Similar to previously reported work, these image features suggest that patients who are likely to develop xerostomia have more locally heterogeneous salivary glands. The heterogeneity differences can be seen in the representative images (Figs. 2 and 3) where patients with xerostomia had more regions of low intensities in both parotid and submandibular glands compared to those patients without xerostomia. This is consistent with previously published work demonstrating that patients who develop xerostomia after RT have more heterogeneous parotid gland tissue [22]. More recently, MR derived image features of the parotid glands were used in the prediction of xerostomia in HNC patients [31]. This important work demonstrated that high signal intensity, specifically the 90th percentile of the MR-intensities in parotid glands improved the performance of the xerostomia prediction model. It is well known that high signal intensity in T1-weighted images is related to fat deposition because of the short T1 relaxation time of fatty tissue [43]. In fact, fat deposition may represent the loss of normal glandular cells as this phenomenon is also seen in diseases such as Sjögren’s syndrome which is characterized by autoimmune destruction of salivary and lacrimal glands [44]. Of note, the salivary glands of patients with Sjögren’s syndrome have also been shown to be more heterogeneous than those without this syndrome [45]. Fatty replaced salivary glands have also been shown to be related to age [46] and xerostomia [47]. However, in our cohort, age was not correlated with image features or xerostomia. On CT, fatty tissue appears as low density [48]. This is consistent with the representative CT images (Figs. 2 and 3) of the parotid and submandibular glands, where the patients with xerostomia had hypodense salivary glands (with obvious local heterogeneous regions).

Finally, in our xerostomia prediction models, for our training cohort, there were no significant differences between our DVH, CT-only, and MR-only models. However, when CT and MR were combined, the performance improved compared to DVH alone. More importantly, we observed that the combination of dosimetry and image features improved overall prediction compared to dosimetry or image features alone. However, the specificity of our models with CT, MR, and DVH-only features was low. In fact, the combination of DVH + CT + MR features did not lead to a significant improvement in sensitivity and specificity. With the addition of all features in a single model, the sensitivity improved only modestly. We should note that majority of our patients did not develop xerostomia, resulting in a biased dataset which could influence sensitivity and specificity. Compared to previously published work evaluating CT image features to predict xerostomia at 12 months [22], the performance of our models was comparable. This work reported an AUC of 0.77 with the inclusion of CT features, specifically features derived from the GLRLM and GLSZM. Other work that has used imaging to predict xerostomia at 12 months using CT only [37, 38] and MR only [31] parotid gland image features has also demonstrated comparable performance to our models (AUC range: 0.60–0.80). Cone beam CT of the parotid glands has also been used to predict xerostomia in a single cohort with performance ranging from 0.71–0.76 [49]. Other work that has used CT parotid image features with dosimetry in a single cohort with nested cross validation has also shown model performances in the range of 0.68–0.88 [50]. In our validation cohort, we observed a similar trend where combining imaging improved performance. Adding dosimetry to our training cohort did improve performance which is consistent with previously published work that has shown the prediction of xerostomia improves when CT image features are combined with dosimetric information [18, 22, 35, 38]. However, in our validation set, adding dosimetry to imaging features did not improve performance. It should be noted that our work used time to separate our training and validation sets. The decrease in performance of the DVH model may be indicative of evolving practices of the attending physicians. Specifically, changes in physician preferences of dose constraints to the salivary glands. The reduction in performance may also reflect limitations of the DVH in capturing 3D spatial information. This may also explain the decrease in performance of the validation models that contained DVH features. It should also be noted that combining clinical data with CT and MR significantly improved xerostomia prediction compared to CT alone. Although the receiver operating characteristic curves had overlapping confidence intervals, there was a trend towards prediction improvement when combining clinical data with dosimetry and image features compared to dosimetry and CT features alone which to our knowledge has not been previously demonstrated. Future work in an independent dataset is required to further determine the benefits of combining imaging modalities in outcome prediction modelling.

Although this study provides promising preliminary results, future work is needed to ascertain the generalizability of these findings. It should be noted that random variation in small datasets can often be mistakenly interpreted as meaningful (i.e. overfitting), and as a consequence the model may not perform as well in independent datasets. In the present work, the risks of overfitting the model were addressed by pre-selecting variables based on their inter-correlation (with no correction for multiple comparisons since *p*-values at this step were simply used to selected a group of candidate features which were further refined using LASSO), cross-validation of the internal dataset, and validating our models using a temporally split dataset [30]. It should be noted that temporal splitting is an intermediate validation method compared to internal and external validation [30]. Future work will need to validate these models on an independent external dataset. The presence of multiple correlated explanatory variables can lead to unstable models with highly variable coefficient estimates and incorrect selection of significant texture features. In this work, collinearity was addressed by determining the Pearson correlation coefficient between two features [30–32]. If the correlation coefficient was larger than 0.80, only the variable with the highest correlation with xerostomia was selected. Modality specific resampling was not performed for the CT images. and non-cubic voxels were used for radiomics analysis, similar to prior studies [18, 22, 31, 38]. Resampling images compared to using the original resolution before feature computation is an active area of radiomics research, and there is no widely accepted recommendation. Resampling images to an isotropic resolution may lead to better interpretation of certain features, but there will be information loss/degradation due to interpolation process. For our MR images, we used the same scanning protocol for training and validation. This may limit the translatability and generalizability of our results because MR intensities are highly dependent on scanning protocol. Also, unlike CT, MR signal-intensity is influenced by hardware factors such as the positioning of the RF coils, which introduce inter-scan variability. Although normalization of MR data has been proposed to address this, the benefits of normalization for radiomic prediction models to differentiate patients with or without xerostomia has not been well established. Future work is needed to establish the benefits of signal normalization for radiomic prediction models of xerostomia. In our work, salivary glands were contoured by the patient’s attending radiation oncologist or by multi-atlas-based auto-segmentation with manual assessment/correction (when clinical contours were not available). Although multiple observers did not contour the same patient, multiple observers’ contours of the glands were included in our feature selection and prediction model building process. Therefore, we anticipate that the selected features are robust to contour variability while being relevant to the outcome. Although previous studies have shown that inter-observer delineation variability has a relevant influence on radiomics analysis [22, 51], we should note that it is important to determine a model that is robust to variability in raw clinically available data so that it can be used in a real clinical scenario. However, further study will be needed to better understand the influence of contour variability to the computed radiomics features and successive feature selection-prediction performance. Finally, we acknowledge that our image feature analysis was limited to a single bin size for CT and single bin count for MRI. Texture features have been shown to be affected by the bin width or number of bins used to discretize image intensities. Although the optimal bin width/count for image feature analysis has not been established, previous HNC work has used a 25 unit bin width (similar to the bin width we used) for the evaluation of image features [27, 28]. However, since image features depend on the way they are computed (i.e. using different binning strategies) further work is needed to investigate the dependency of bin width and the selection of image features on xerostomia prediction.

## Conclusions

This study suggests that baseline image features stemming from both the parotid and submandibular glands have the potential to be used as a clinical surrogate for baseline function. Features from the submandibular glands, specifically, may provide insight into unstimulated salivary function thus providing an improved prediction of susceptibility to post-RT xerostomia. Although there was a trend towards prediction improvement when all data was combined, future work is required to further determine the benefits of combining imaging modalities in xerostomia prediction. Taken together, prediction models based on these features can further our understanding of the development of radiation-induced xerostomia and allow us to develop patient-specific adaptations to radiation treatment plans to minimize toxicity.

## Additional files

Additional file 1: **Figure S1.** Training set receiver operating characteristic (ROC) curves shown for: a) DVH Model, d) CT Model, c) MR Model, d) CT + MR Model, e) DVH + CT Model, f) DVH + CT + MRI Model, g) Clinical+CT + MR Model, and h) Clinical+DVH + CT + MR Model. Gray identifies the 95% confidence interval (CI). (TIF 449 kb)

Additional file 2: **Figure S2.** Test set receiver operating characteristic (ROC) curves shown for: a) DVH Model, d) CT Model, c) MR Model, d) CT + MR Model, e) DVH + CT Model, f) DVH + CT + MRI Model, g) Clinical+CT + MR Model, and h) Clinical+DVH + CT + MR Model. Gray identifies the 95% confidence interval (CI). (TIF 407 kb)

Additional file 3: **Table S1.** GLM summary including odds ratios (OR) and 95% confidence interval (CI) for the prediction of xerostomia. (DOCX 21 kb)

## Data Availability

The datasets used and analyzed during the current study are available from the corresponding author on reasonable request.
