# Supplementary figures and images for: Predicting acute radiation induced xerostomia in head and neck Cancer using MR and CT Radiomics of parotid and submandibular glands
Source: Radiat Oncol. 2019 Jul 29;14:131. doi: 10.1186/s13014-019-1339-4 (PMC6664784; doi:10.1186/s13014-019-1339-4)

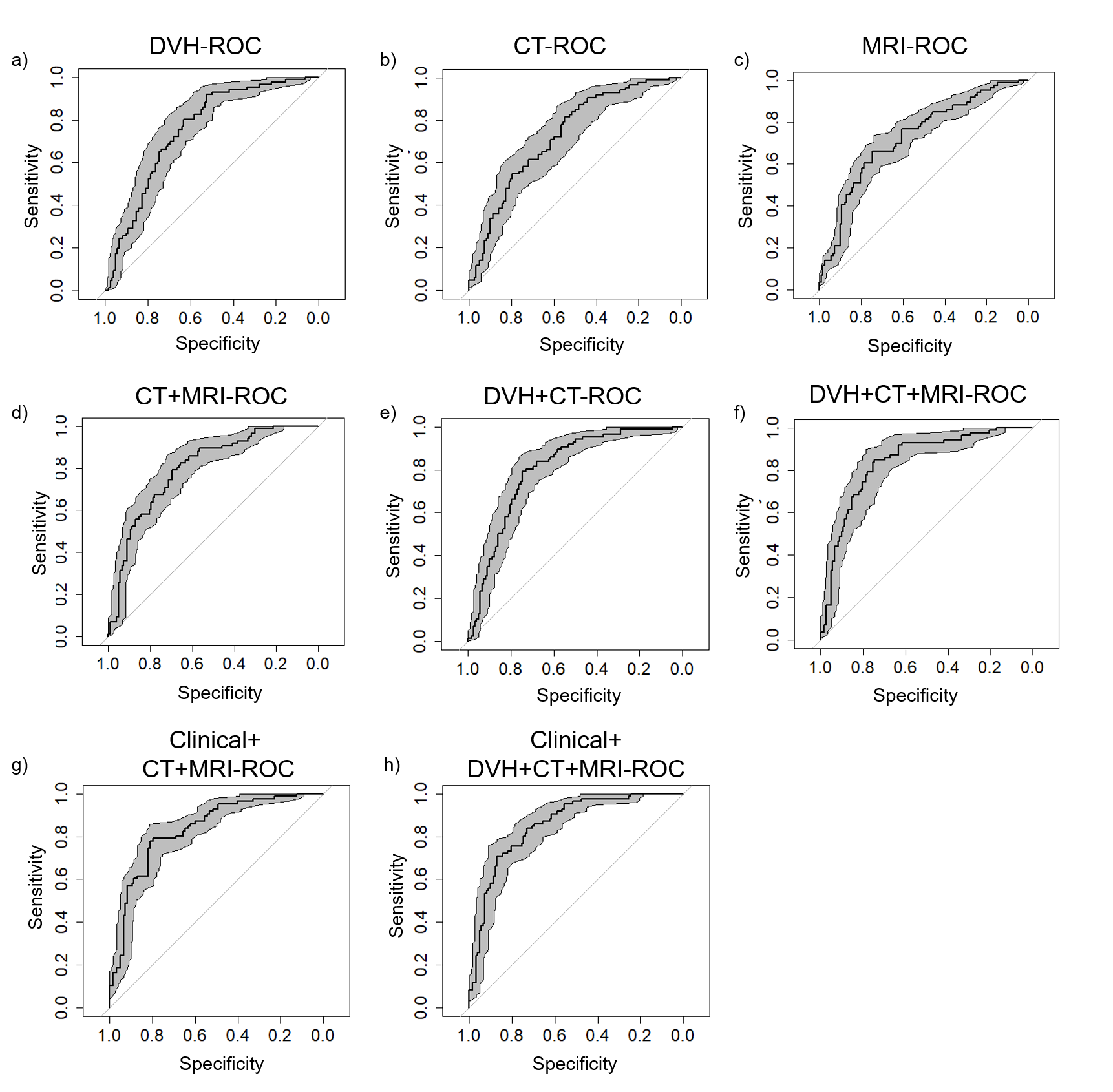

Supplement: Supplementary file 1 — Figure S1. Training set receiver operating characteristic (ROC) curves shown for: a) DVH Model, d) CT Model, c) MR Model, d) CT + MR Model, e) DVH + CT Model, f) DVH + CT + MRI Model, g) Clinical+CT + MR Model, and h) Clinical+DVH + CT + MR Model. Gray identifies the 95% confidence interval (CI). (TIF 449 kb) [file 13014_2019_1339_MOESM1_ESM.tif]

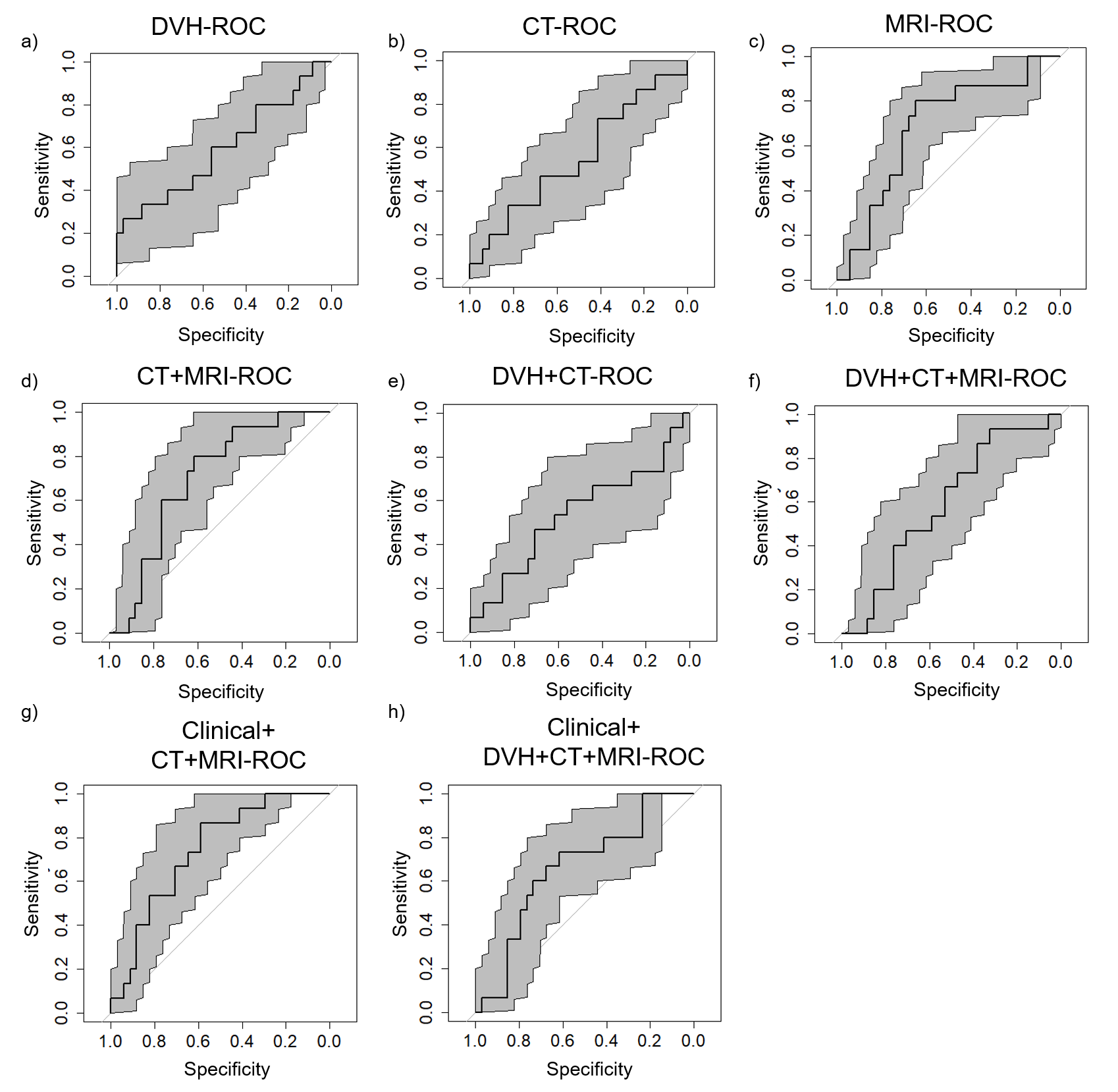

Supplement: Supplementary file 2 — Figure S2. Test set receiver operating characteristic (ROC) curves shown for: a) DVH Model, d) CT Model, c) MR Model, d) CT + MR Model, e) DVH + CT Model, f) DVH + CT + MRI Model, g) Clinical+CT + MR Model, and h) Clinical+DVH + CT + MR Model. Gray identifies the 95% confidence interval (CI). (TIF 407 kb) [file 13014_2019_1339_MOESM2_ESM.tif]
