# Supplementary material for: Predicting acute radiation induced xerostomia in head and neck Cancer using MR and CT Radiomics of parotid and submandibular glands
Source: Radiat Oncol. 2019 Jul 29;14:131. doi: 10.1186/s13014-019-1339-4 (PMC6664784; doi:10.1186/s13014-019-1339-4)
Supplement: Supplementary file 3 — Table S1. GLM summary including odds ratios (OR) and 95% confidence interval (CI) for the prediction of xerostomia. (DOCX 21 kb) [file 13014_2019_1339_MOESM3_ESM.docx]

**Additional file 3: Table S1.** GLM summary including odds ratios (OR) and 95% confidence interval (CI) for the prediction of xerostomia.

|  | **CT+MR** | | **DVH+CT** | | **DVH+CT+MRI** | | **Clinical+CT+MR** | |
| --- | --- | --- | --- | --- | --- | --- | --- | --- |
|  | **β** | **OR**  **(95% CI)** | **β** | **OR**  **(95% CI)** | **β** | **OR**  **(95% CI)** | **β** | **OR**  **(95% CI)** |
| Intercept | **-5.79** |  | **-6.17** |  | **-4.72** |  | **-6.09** |  |
| Age | - | - | - | - | - | - | -1.06 | 0.34  (0.31-0.38) |
| Gender | - | - | - | - | - | - | 1.03 | 2.83  (1.86-3.80) |
| Tumor Volume | - | - | - | - | - | - | 0.48 | 1.60  (1.60-1.61) |
| cPG D40 | - | - | **1.11** | **3.03**  **(3.02-3.04)** | **1.13** | **3.10**  **(3.09-3.11)** | - | - |
| cSG D60 | - | - | **0.85** | **2.34**  **(2.33-2.35)** | 0.77 | 2.09  (2.08-2.10) | - | - |
| CT cSG wavelet LLL GLSZM Gray Level Non Uniformity Normalized | **0.99** | **2.69**  **(-12.3-17.7)** | **0.77** | **2.17**  **(-12.9-17.2)** | **0.77** | **2.17**  **(-14.1-18.4)** | **1.00** | **2.72**  **(-12.98-18.43)** |
| CT iPG original GLSZM Low Gray Level Zone Emphasis | -0.58 | 0.56  (-9.56-10.7) | -0.55 | 0.58  (-9.07-10.2) | -0.47 | 0.63  (-9.82-11.1) | -0.76 | 0.47  (-10.4-11.34) |
| CT iSG wavelet HLL GLCM Inverse Variance | 0.60 | 1.82  (-18.7-22.2) | 0.18 | 1.20  (-19.8-22.2) | 0.22 | 1.24  (-21.5-23.9) | 0.66 | 1.93  (-19.3-23.1) |
| CT cPG wavelet LHL Total Energy | 0.37 | 1.45  (1.44-1.46) | 0.17 | 1.18  (1.17-1.19) | 0.21 | 1.24  (1.23-1.25) | 0.44 | 1.54  (1.54-1.56) |
| CT iSG wavelet HLL GLRLM Long Run High Gray Level Emphasis | -0.83 | 0.44  (0.43-0.45) | -0.79 | 0.45  (0.44-0.46) | -0.84 | 0.43  (0.42-0.43) | -0.69 | 0.50  (0.49-0.51) |
| CT iPG original first order 10 Percentile | -0.44 | 0.65  (0.64-0.66) | -0.65 | 0.52  (0.51-0.53) | -0.46 | 0.63  (0.62-0.64) | -0.34 | 0.70  (0.69-0.72) |
| CT cPG wavelet LHL GLRLM Long Run High Gray Level Emphasis | 0.42 | 1.52  (1.51-1.53) | 0.56 | 1.75  (1.74-1.76) | 0.47 | 1.60  (1.59-1.61) | 0.43 | 1.53 (1.53-1.54) |
| MR cPG shape Least Axis Length | 0.44 | 1.55  (1.48-1.63) | - | - | 0.40 | 1.50  (1.41-1.58) | **0.94** | **2.54**  **(2.46-2.64)** |
| MR iSG wavelet LHH GLSZM Gray Level Non Uniformity Normalized | **-0.94** | **0.39**  **(-30.0-30.8)** | - | - | -0.75 | 0.47  (-31.6-32.5) | **-1.17** | **0.31**  **(-32.83-33.45)** |
| MR iSG wavelet LHH GLSZM Small Area High Gray Level Emphasis | **1.08** | **2.94**  **(2.93-2.96)** | - | - | **1.24** | **3.45**  **(3.43-3.47)** | **1.19** | **3.30**  **(3.28-3.32)** |
| MR iPG wavelet LHL GLSZM Small Area High Gray Level Emphasis | **-0.79** | **0.45**  **(0.43-0.47)** | - | - | **-0.77** | **0.46**  **(0.44-0.48)** | -0.73 | 0.48  (0.46-0.50) |
| MR iSG wavelet LLH GLSZM Size Zone Non Uniformity Normalized | -0.47 | 0.62  (-10.6-11.9) | - | - | -0.30 | 0.74  (-11.5-12.9 | -0.54 | 0.58  (-11.64-12.80) |

iPG: ipsilateral parotid gland, cPG: contralateral parotid gland, iSG: ipsilateral submandibular gland, cSG: contralateral submandibular gland, GLCM: gray level co-occurrence matrix, GLSZM: gray level size zone matrix, GLRLM: gray level run length matrix; bold indicates significant values (p<.05)
